# Inhibitory activity against carbonic anhydrase IX and XII as a candidate selection criterion in the development of new anticancer agents

**DOI:** 10.1080/14756366.2020.1801674

**Published:** 2020-08-04

**Authors:** Mikhail Krasavin, Stanislav Kalinin, Tatiana Sharonova, Claudiu T. Supuran

**Affiliations:** a Institute of Chemistry, St. Petersburg State University, St. Petersburg, Russia; b Neurofarba Department, Section of Pharmaceutical Sciences, University of Florence, Florence, Italy

**Keywords:** Carbonic anhydrase inhibitors, cancer-related IX and XII isoforms, anticancer agents, screening funnel, enrichment factor

## Abstract

Analysis of the literature data reveals that while inhibition of cancer-related carbonic anhydrase IX and XII isoforms continues to be an important enrichment factor for designing anticancer agent development libraries, exclusive reliance on the *in vitro* inhibition of these two recombinant isozymes in nominating candidate compounds for evaluation of their effects on cancer cells may lead not only to identifying numerous compounds devoid of the desired cellular efficacy but also to overlooking many promising candidates which may not display the best potency in biochemical inhibition assay. However, SLC-0111, now in phase Ib/II clinical trials, was developed based on the excellent agreement between the *in vitro*, *in vivo* and more recently, in-patient data.

## Introduction

Tumour growth and proliferation are strongly associated with hypoxic stress due to poor vascularisation and oxygen deprivation of neoplastic tissues^1^. Adaptive metabolic changes observed in cancer cells include elevated production of acidic metabolites which leads to tumour acidosis^2–4^. Human carbonic anhydrase (*h*CA) isoforms IX and XII are crucial effectors that regulate extracellular pH thus mediating cancer cell proliferation, invasion, and metastasis^5^^,^^6^. These membrane-bound proteins are zinc metalloenzymes that catalyse the reversible hydration of CO_2_ to bicarbonate anion and proton on cell surface (Equation (1))^7^. Many studies confirmed *h*CA IX/XII upregulation in hypoxic tumours^8^^,^^9^. In particular, *h*CA IX which has limited expression in normal tissues, is a marker of aggressive and drug-resistant cancer cell phenotypes indicating poor prognosis for patients^10^. Arguably, *h*CA XII is widely distributed in the human body and considered a marker for less malignant tumours^11^. Targeting the catalytic activity of the cell surface carbonic anhydrases has been and continues to be considered a promising therapeutic approach in the antineoplastic field, either for suppressing tumour growth or overcoming drug resistance of cancer cells^12^^,^^13^.

(1) $${\text{CO}}_{2}+{\text{H}}_{2}\text{O}⇄{\text{HCO}}_{3}^{-}+{\text{H}}^{+}$$

**Figure 1.**
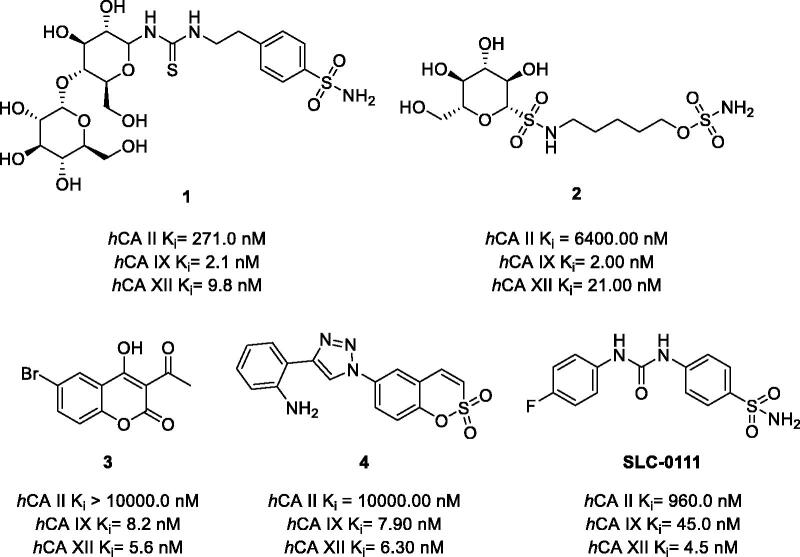
Potent and selective *h*CA IX/XII inhibitors from among sulphonamides (**1**), sulfamates (**2**), coumarins (**3**), and sulfocumarins (**4**) and SLC-0111 with its CA inhibition profile.

Much progress has been made in the last decade towards anticancer agents based on small-molecule inhibitors of *h*CA IX and/or XII^14^^,^^15^. These efforts typically involved screening of compound libraries in search for potent and selective blockers of these isozymes, followed by detailed *in vitro* and *in vivo* characterisation of the most promising candidates. Many new compounds have been thus identified capable of inhibiting recombinant *h*CA IX and/or XII in the low nanomolar to subnanomolar range with remarkable selectivity over other carbonic anhydrase isoforms. These chemotypes often contained a primary sulphonamide or sulfamate (e.g. **1** and **2**) group or were based on a coumarin and sulfocoumarin core (such as **3** and **4**, respectively)^16–19^. Of these classes, ureido-substituted benzene sulphonamides (USBs) made the most progress with **SLC-0111** recently entering phase Ib/II clinical trials (Figure 1)^20^^,^^21^.

Despite significant advances, the discovery of *h*CA-targeted anticancer agents is not a straightforward endeavour, with many aspects remain unclear. In fact, our own efforts as well as those reported in the literature often yield structures, which display frustratingly modest antiproliferative effect, although possessing profound inhibition of isolated *h*CA IX/XII. Such inconsistencies occurring between the ability of some *h*CA inhibitors to block the recombinant enzyme catalytic function and their effects on cancer cells constitute a challenge that has been much less addressed, in comparison to those related to the SAR and selectivity studies^22–24^. Thus, although the potential of *h*CA IX and XII as anticancer targets is supported by much evidence, including the well-documented efficacy of *h*CA-inhibitory antibodies *in vitro* and *in vivo*, the success rate of small-molecule inhibitors upon the transition to cell culture setting is still below the desirable level^18^^,^^25–29^. Taking this into account, attempts to re-evaluate the conventional drug discovery workflow that has existed in this field are of importance. Indeed, while factors affecting the activity in cells are poorly understood, the drug-discovery funnel beginning with recombinant protein-based profiling should be critically evaluated. Therefore, we undertook a literature survey and analysis regarding possible disconnects for *h*CA inhibitors, leading to a dramatic change of the compound’s activity upon the transition from the recombinant isolated protein to the cell-based models.

## Discussion

Disappointingly, a large fraction of publications on the subject reported compounds’ cytotoxicity data obtained in normoxic conditions thus making the involvement of *h*CA IX and XII debateable^30–45^. Although such testing can be relevant in some cases, we consider these conditions irrelevant as long as antiproliferative activity of *h*CA inhibitors is concerned. This is primarily due to limited expression of the target isoforms, if any, reported for many cancer cell lines under these conditions^8^^,^^46^. The studies which employ normoxic MCF-10A cells lacking *h*CAIX/XII on their surface are illustrative^47^. Furthermore, some investigations involved cell lines displaying robust *h*CA IX activity in the cytosol, such as MDA-MB-231 and MCF-7^48^. The said non-default localisation of *h*CA IX could lead to overestimation of the protein expression and activity on the cell membrane, thus leading to the problematic interpretation of the data obtained^16^. Thus, the choice of experimental conditions often renders the results ambiguous, making them rather difficult to analyse.

**Figure 2.**
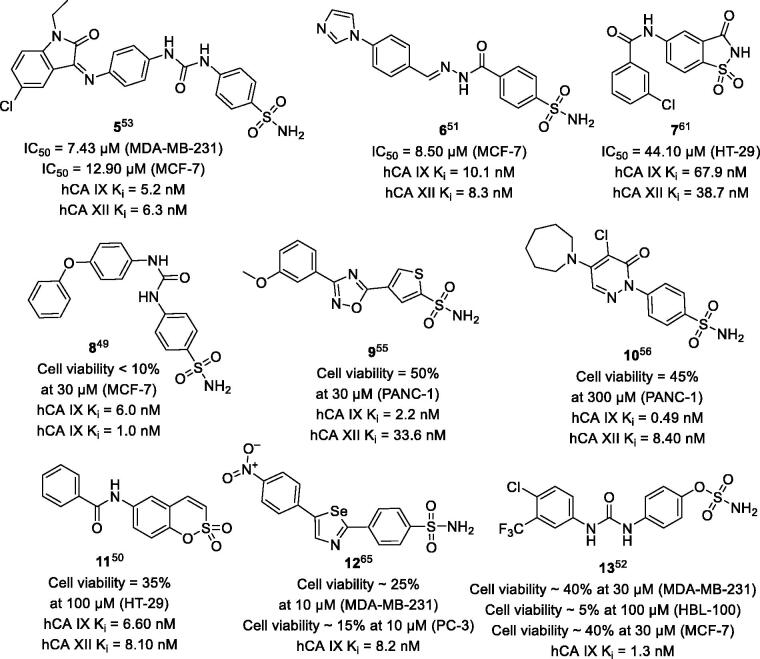
Potent *h*CA IX/XII inhibitors displaying good translation of their activity into antiproliferative activity against cancer cells under hypoxia.

To our delight, however, there is a large number of mechanistically relevant experiments reported in the literature which address the ability of *h*CA IX/XII inhibitors to block the growth of hypoxic cancer cells overexpressing the target isozymes^49–67^. Moreover, a certain cohort of studies revealed compounds possessing both favourable *h*CA inhibitory profile and significant anticancer activity, as exemplified by structures **5**–**13** (Figure 2)^49–56^^,^^58–61^^,^^64–67^. In fact, quite a number of single-digit nanomolar *hCA* IX/XII inhibitors significantly suppressed cancer cell growth. Additional data underscoring the specificity of this antiproliferative action in cancer over normoxic and non-cancerous cells highlight the potential of these findings for practical applications. Meanwhile, the drug-like character of some frontrunners makes them intriguing starting points for medicinal chemistry optimisation. Once selected based on their *h*CA inhibitory profiles, these compounds might be useful indeed in designing antineoplastic drugs either of combinative or single-agent use^49–53^^,^^55^^,^^56^^,^^61^^,^^65^.

**Figure 3.**
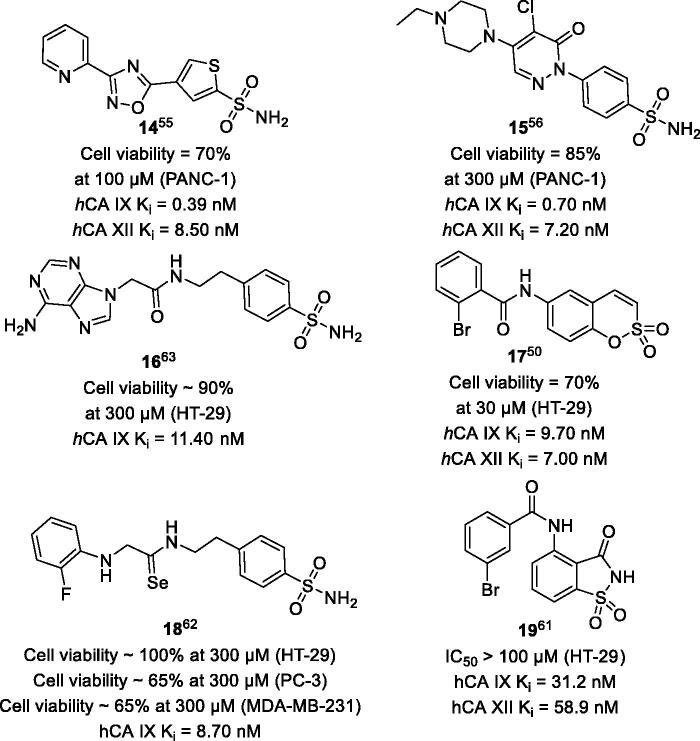
Potent *h*CA IX/XII exhibiting poor antiproliferative activity under hypoxic conditions.

In contrast to the aforementioned examples of good correlation between *h*CA IX/XII inhibition and cytotoxicity against cancer cells in hypoxic conditions, the reverse is true for a strikingly large number of examples^50^^,^^55–57^^,^^60–63^^,^^67^. In these cases, many potent *h*CA IX/XII inhibitors, such as **14**–**19**, did not possess a substantial activity against cancer cells. Furthermore, these examples often share structural similarity with the hit compounds originating from the same screening series (cf. examples in Figure 2). Despite the obvious resemblance, no effect observed under identical conditions, again, indicates our limited understating of the factors influencing *h*CA inhibitors activity in the intact cells (Figure 3)^50^^,^^55^^,^^56^^,^^61–63^. According to the existing discovery workflow in this field, if these compounds are selected for the detailed evaluation based solely on their enzyme inhibitory profile, this could have diverted the investigator’s attention from potentially efficacious anticancer agents.

**Figure 4.**
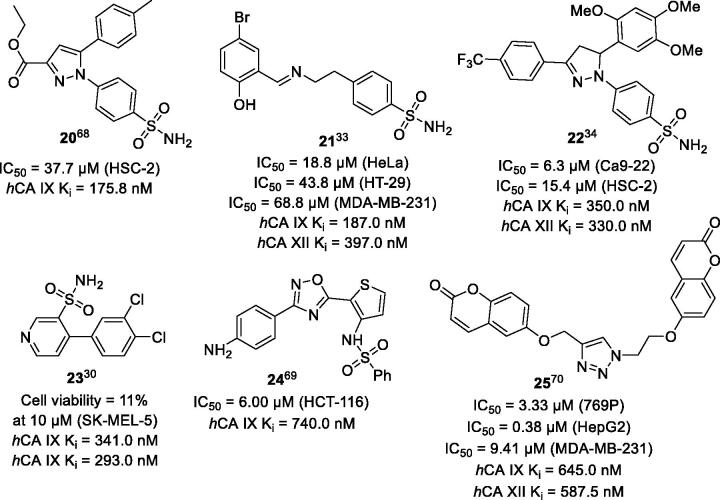
Sulphonamides and coumarins demonstrating potent antiproliferative properties despite being weak inhibitors of the recombinant *h*CA IX/XII.

A large portion of compounds may be overlooked in the anticancer agent development projects because of their moderate inhibitory properties towards *h*CA IX/XII cancer-related isozyme duo. This fairly populated category of compounds possessing moderate to low inhibitory properties towards recombinant *h*CA IX/XII features many compounds that are based on privileged chemotypes (e.g. **20**–**25**). Evidently, in most studies, these relatively inactive molecules would have not been progressed based solely on their inhibitory profile. At the same time, several reports reveal that and unexpectedly good cytotoxicity towards cancer cells can be demonstrated by such compounds (Figure 4)^30^^,^^33^^,^^34^^,^^68–70^.

These occasional findings supported the questioning of the direct predictive power of *h*CA IX/XII inhibitory profile for a compound’s anticancer activity. Understanding the significance of the candidate molecule prioritisation problem in question is essential while ignoring it may lead to many promising compounds being overlooked in want of more potent *h*CA IX/XII inhibitors. Aiming to draw a more informative picture, we have graphically represented the relationship between the anticancer effect (in this case, irrespective of the specific experimental conditions employed in various studies) and the *h*CA IX/XII inhibitory activity of the compounds^30–45^^,^^49–58^^,^^71–74^. In order to summarise the somewhat heterogeneous literature data, we have visualised the reported antiproliferative agents in the plots with the *Y*-axis displaying *K*_i_’s against *h*CA IX (Figures S1 and S2) and *h*CA XII (Figures S3 and S4). Several groups of compounds were distinguished and marked by a different colour with regard to their effect on cancer cells: (1) <50% cell survival at 10 µM (or IC_50_=0.1–10 µM), green; (2) <50% cell survival at 50 µM (or IC_50_=11–50 µM), blue; (3) IC_50_ 51–100 µM, tangerine; (4) IC_50_=101–150 µM, cyan; (5) IC_50_=151–200 µM, magenta; (6) IC_50_ >200 µM, red; (7) <50% cell growth inhibition at 10 or 50 µM, yellow. As is evident from Figures S1–S4, potent cancer cell growth suppressors can possess vastly different levels of inhibitory activity against *h*CA IX/XII. However, the low nanomolar region of *K*_i_ values appears to be most populated with compound possessing strong effect on cancer cells (Figures S1 and S3). Meanwhile, a fairly large portion of compounds did not possess noticeable antiproliferative activity while displaying various levels of *K*_i_ values towards recombinant *h*CAs (Figures S2 and S4). The prevailing occurrence of these compounds in the nanomolar range of *h*CA IX/XII inhibitory activity is likely the result of the currently applied drug discovery funnel where the best *h*CA IX/XII inhibitors are primarily selected for subsequent evaluation for their effects on cancer cells.

The supremacy of *in vitro* potency for drug discovery decision making has been much debated with respect to different fields of medicinal chemistry^75^. In complex biological environment, multiple variables such as ligand residence time or metabolic stability^76^^,^^77^ can determine the observed efficacy. However, the said parameters are not routinely looked at when screening for potential *h*CA IX/XII inhibitors. While tens to hundreds nanomolar levels of on-target activity prevail among clinically used drugs^77^, such activity levels are easily achievable for primary sulphonamides *h*CA IX and\or XII isoforms, as can be deduced from Figures S1–S4. This gives us an idea of a different approach to the discovery of anticancer agents based on *h*CA IX/XII inhibition. Considering, that multiple factors appear to be at play when cytotoxicity towards cancer cells is concerned, front-loading *h*CA IX/XII inhibition as a candidate selection criterion should be taken with caution. *In vitro* screening is indispensable for identifying chemotypes with a clear tendency to inhibit the target isozymes. Once identified and expanded into larger, SAR-informative follow-on libraries, these candidate chemotypes should perhaps be screened primarily against cancer cells so as not to overlook promising candidates which may not necessarily be endowed with the best *h*CA IX/XII inhibitory potency.

## Conclusions

Literature data indicate that inhibition of the recombinant protein possesses a somewhat limited predictive power for the compounds’ ability to block cancer cell growth. Not questioning the role of the cell surface *h*CA isoforms in tumour growth and survival, this conclusion, however, highlights the imperfection of the existing approach to the design and discovery of antineoplastic drugs in this field. In fact, by selecting compounds for *in vitro* and *in vivo* characterisation based solely on their inhibitory profile, one could discard many promising hit-structures. Resources expended for optimising molecules’ potency and selectivity towards the target recombinant protein may also be wasted as they do not necessarily ensure antiproliferative activity. On the other hand, a wealth of positive examples in the literature of a strong correlation between *h*CA IX/XII inhibition and cytotoxicity towards cancer cells indicates that demonstrated tendency of new chemotypes to inhibit these isoforms can serve as a robust enrichment factor for designing screening libraries that target cancer cells. Moreover, detailed SAR studies of different carbonic anhydrase inhibitors with regard to *h*CA IX and/or XII are of importance due to the significance of developing new ligands of these targets for tumour imaging, drug delivery and other purposes^78^.

## Supplementary Material

Supplemental Material
