# Supplementary material for: Inhibitory activity against carbonic anhydrase IX and XII as a candidate selection criterion in the development of new anticancer agents
Source: J Enzyme Inhib Med Chem. 2020 Aug 4;35(1):1555–61. doi: 10.1080/14756366.2020.1801674 (PMC7470080; doi:10.1080/14756366.2020.1801674)

Supplementary material for

Inhibitory activity against carbonic anhydrase IX and XII as a candidate selection criterion in the development of new anticancer agents

Mikhail Krasavin,[a] Stanislav Kalinin, Tatiana Sharonova and Claudiu Supuran*

Figure S1. The relationship between compound's K_i toward *h*CA IX and their ability to suppress cancer cell growth («active» subset)

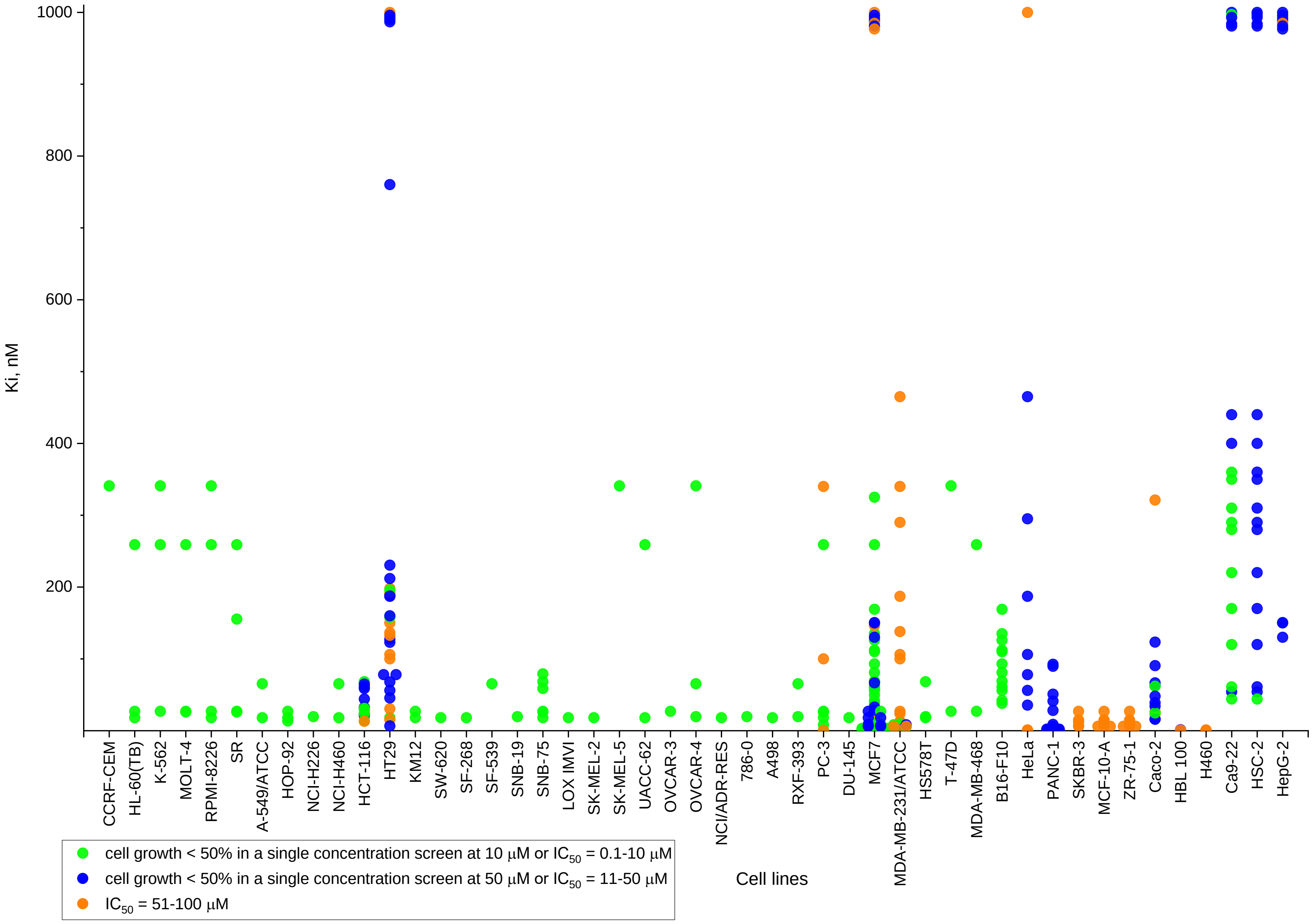

Figure S2. The relationship between compound's K_i toward *h*CA IX and their ability to suppress cancer cell growth («inactive» subset)

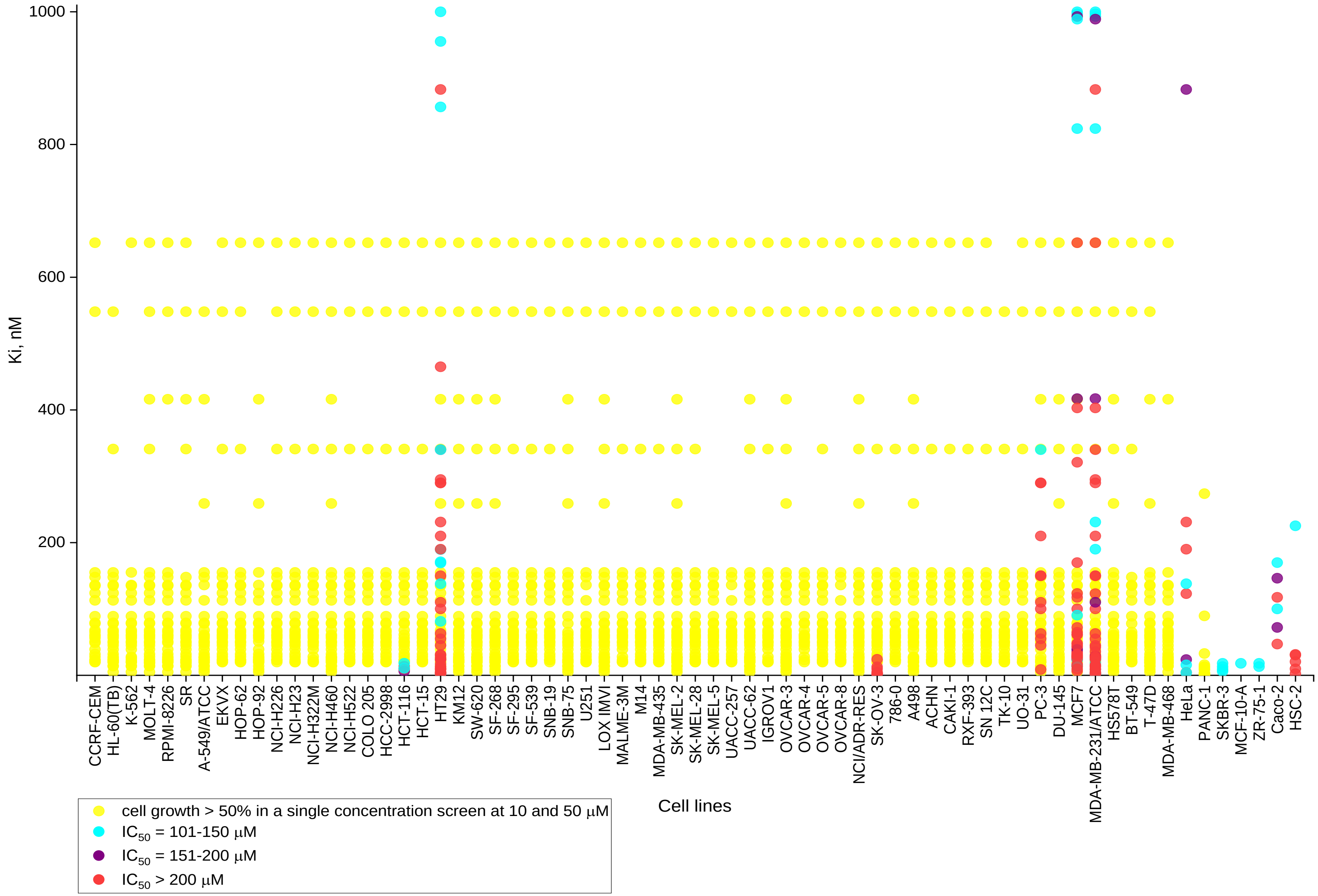

Figure S3. The relationship between compound's K_i toward *h*CA XII and their ability to suppress cancer cell growth («active» subset)

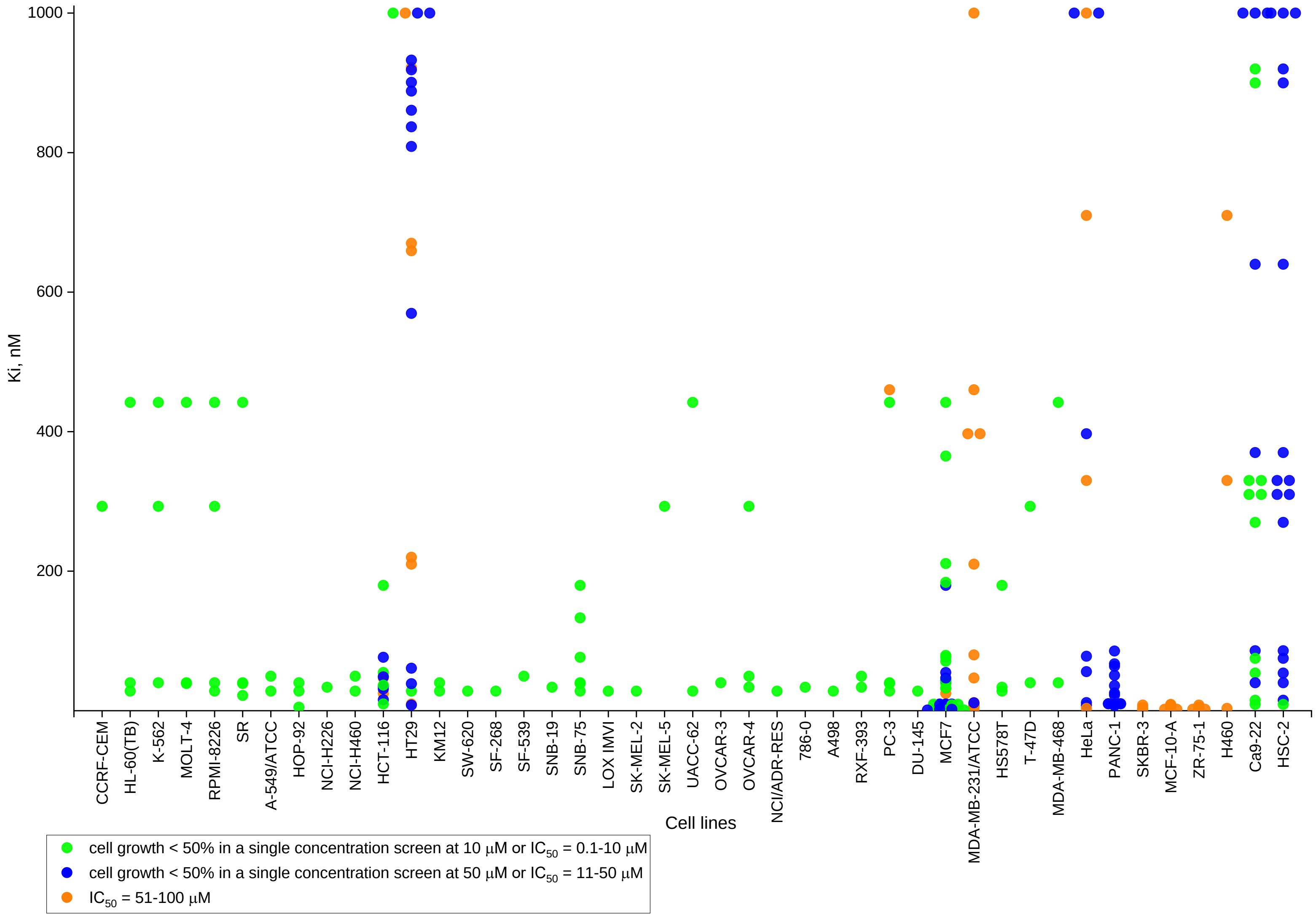

Picture 4. The relationship between compound's K_i toward *h*CA XII and their ability to suppress cancer cell growth («inactive» subset)

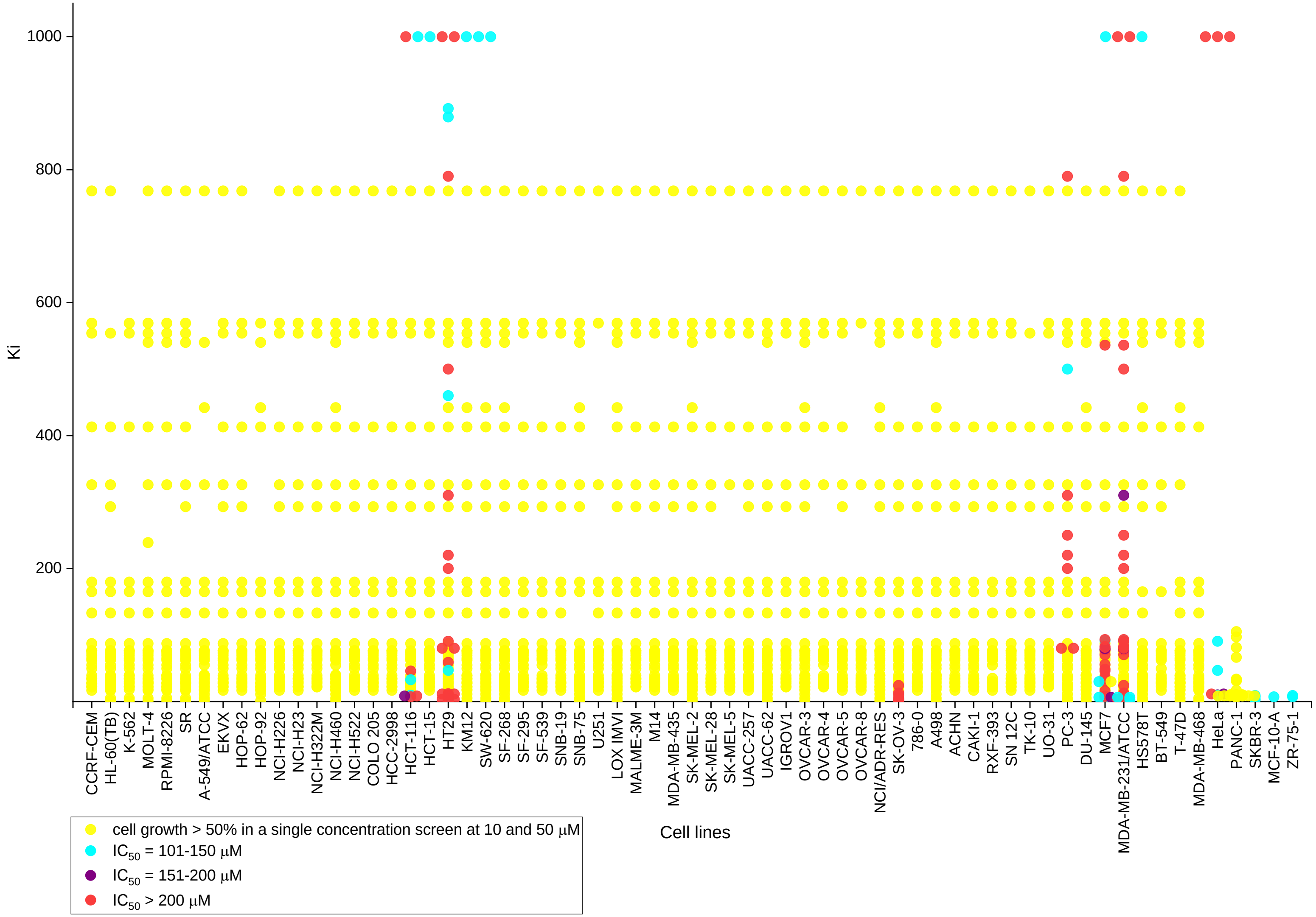

Supplement: Supplemental Material [file IENZ_A_1801674_SM1387.pdf]
